# Can artificial intelligence detect type 2 diabetes in women by evaluating the pectoral muscle on tomosynthesis: diagnostic study

**DOI:** 10.1186/s13244-024-01661-4

**Published:** 2024-03-27

**Authors:** Meltem M. Yashar, Ilayda Begum Izci, Fatma Zeynep Gungoren, Abdulkadir A. Eren, Ali A. Mert, Irmak I. Durur-Subasi

**Affiliations:** 1 Faculty of Medicine, Istanbul Medipol University, Istanbul, Turkey; 2 Deloitte, Istanbul, Turkey; 3 Department of Radiology, Florence Nightingale Hospital, Istanbul, Turkey; 4 Faculty of Medicine, Department of Radiology, Istanbul Medipol University, Istanbul, Turkey; 5 Faculty of Medicine, Department of Internal Medicine, Istanbul Medipol University, Istanbul, Turkey; 6 International Faculty of Medicine, Department of Radiology, Istanbul Medipol University, Istanbul, Turkey

**Keywords:** Artificial intelligence, Diabetes mellitus, Digital breast tomosynthesis, Glycosylated hemoglobin A1c, Pectoral muscle

## Abstract

**Objectives:**

This retrospective single-center analysis aimed to evaluate whether artificial intelligence can detect type 2 diabetes mellitus by evaluating the pectoral muscle on digital breast tomosynthesis (DBT).

**Material method:**

An analysis of 11,594 DBT images of 287 consecutive female patients (mean age 60, range 40–77 years) was conducted using convolutional neural networks (EfficientNetB5). The inclusion criterion was left-sided screening images with unsuspicious interpretation who also had a current glycosylated hemoglobin A1c (HBA1c) % value. The exclusion criteria were inadequate imaging, history of breast cancer, and/or diabetes mellitus. HbA1c values between 5.6 and 6.4% were categorized as prediabetic, and those with values ≥ 6.5% were categorized as diabetic. A recorded HbA1c ≤ 5.5% served as the control group. Each group was divided into 3 subgroups according to age. Images were subjected to pattern analysis parameters then cropped and resized in a format to contain only pectoral muscle. The dataset was split into 85% for training and 15% for testing the model’s performance. The accuracy rate and F1-score were selected as performance indicators.

**Results:**

The training process was concluded in the 15th epoch, each comprising 1000 steps, with an accuracy rate of 92% and a loss of only 0.22. The average specificity and sensitivity for all 3 groups were 95%. The F1-score was 0.95. AUC-ROC was 0.995. PPV was 94%, and NPV was 98%.

**Conclusion:**

Our study presented a pioneering approach, applying deep learning for the detection of diabetes mellitus status in women using pectoral muscle images and was found to function with an accuracy rate of 92%.

**Critical relevance statement:**

AI can differentiate pathological changes within pectoral muscle tissue by assessing radiological images and maybe a potential diagnostic tool for detecting diabetes mellitus and other diseases that affect muscle tissues.

**Key points:**

• AI may have an opportunistic use as a screening exam for diabetes during digital breast tomosynthesis.

• This technique allows for early and non-invasive detection of diabetes mellitus by AI.

• AI may have broad applications in detecting pathological changes within muscle tissue.

**Graphical Abstract:**

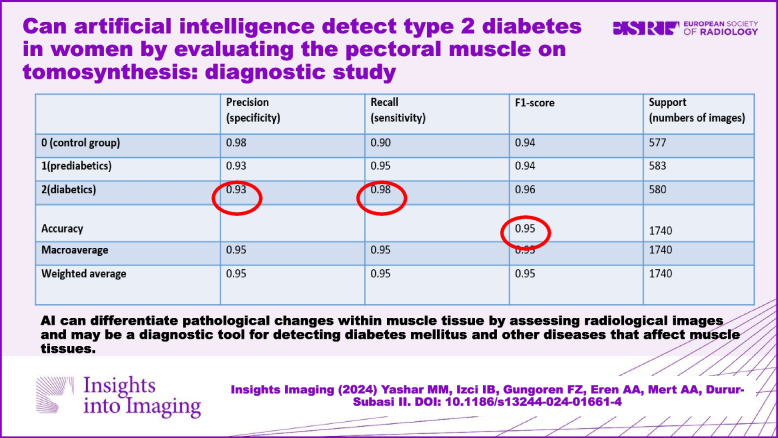

## Introduction

According to 2021 statistics, the global prevalence of diabetes mellitus is 10% among people aged 21–79 age [1]. Due to the emphasis on nutrition around ready-made and fast foods, sedentary lifestyle, and insufficient exercise habits, the prevalence of type 2 diabetes mellitus (T2DM) continues to rise worldwide. The use of glucose in tissues that produce energy through the mitochondria is limited. When the body cannot use glucose efficiently, it resorts to alternative energy production pathways, leading to secondary ectopic fat accumulation.

T2DM is associated with microvascular complications such as nephropathy, neuropathy, and neglected retinopathy, which are related to higher mortality. Additionally, macrovascular complications have been substantially associated with death rate (HR = 2.00, 95% CI = 1.69–2.38) [2]. In addition to traditional micro- and macrovascular disease leading to considerable disability, “frailty and sarcopenia” are emerging as a third category of complications [3]. Loss of skeletal muscle mass, muscle weakness, and/or loss of physical function are the hallmarks of the syndrome known as sarcopenia [4]. Insulin resistance found in T2DM is an indirect contributing factor for diminished physical function and mobility, and T2DM is linked with a threefold increased risk of sarcopenia, as evidenced by 8.2% of newly diagnosed T2DM patients having sarcopenia [5].

Skeletal muscles are the largest and most insulin-sensitive organs in the body. This enables them to be responsible for the majority of glucose uptake from the blood. They therefore play a key role in regulating glucose homeostasis. Approximately 80–90% of glucose consumption and myokine synthesis occur in striated muscles [6]. Good-quality muscle tissue, defined by the normal attenuated muscle area/total abdominal muscle area ratio, has protective effects against T2DM [7]. A cohort study discovered that in previously healthy people, muscle mass was inversely correlated with the incidence of T2DM [8]. Ultrasound images of the deltoid muscles of patients with T2DM were recorded as hyperechoic compared with healthy individuals of the same age group [9]. This is a promising sign that the ability of artificial intelligence (AI) to analyze image features, such as shape, volume, and area, can be used to detect diabetic status. Deep learning, a recent innovation in AI, has shown the ability to, for some applications, interpret medical images with sensitivities and specificities at or near that of skilled clinicians [10]. However, there is currently no AI model attempting to diagnose diabetes mellitus based on radiological muscle tissue images.

Digital breast tomosynthesis (DBT) is a screening method for breast cancer in patients over 40 years of age. The pectoral muscle is included in the imaging to ensure that the breast tissue is covered. Although there are significant AI studies related to DBT, they have focused on the benign–malignant distinction, and there has been no previous scenario in which DBT has been used as an opportunistic diagnostic tool. From a physiological perspective, at the age of 40, progressive and generalized loss of muscle mass can be observed [11]. DM has been proven to negatively contribute to this process. Accordingly, our study’s focus group was more vulnerable to the effects of diabetes mellitus on the skeletal muscles. Early diagnosis of T2DM can be lifesaving and reduce the burden of health costs. It can also prevent complications, improve patients’ quality of life, and reduce the time and budget spent on treating complications, thus reducing the workload of doctors.

Although routine practice uses blood biochemistry for the diagnosis of DM, visceral fat and muscle mass may also be good predictors of T2DM [8, 12]. Therefore, in this study, we aimed to investigate the pectoral muscle, which determines the quality of DBT and is not currently used for any output regarding the muscle tissue. We have developed a deep learning AI model that detects patients’ diabetes mellitus status from the muscle tissue within the imaging area, as a non-invasive and alternative method to blood biochemistry.

## Material/method

This retrospective observational diagnostic study was approved by the non-interventional clinical research ethics committee at Medipol University with reference number E-10840098-772.02-1232 (17,02.2022/decision number:187). This was a retrospective, anonymized diagnostic study, ensuring patient privacy and confidentiality. Therefore, informed consent could not be acquired nor was it required.

### Subjects

**Fig. 1:**
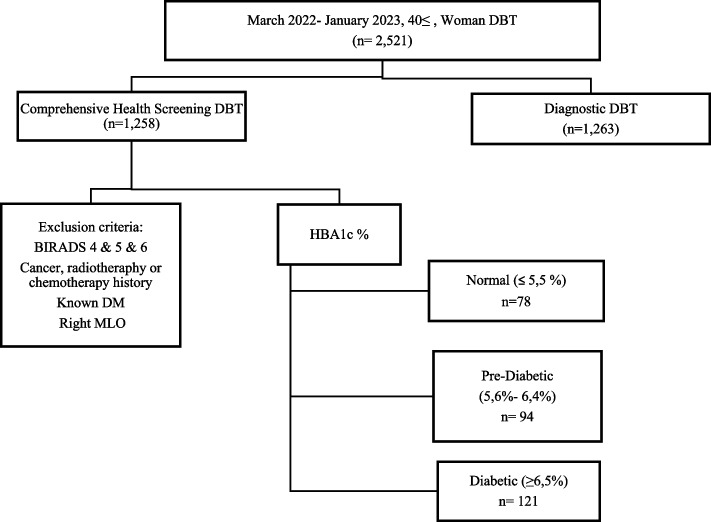
Data selection flowchart

Our electronic records between March 2022 and January 2023 were analyzed. Asymptomatic patients with screening tomosynthesis and same-day HbA1c% values were selected. A retrospective analysis was conducted on 2521 DBT examinations of women aged 40 years or older who underwent breast tissue screening via DBT at the Breast Imaging Unit of the Department of Radiology at Medipol Mega University Hospital. All patients (all females; mean age ± standard deviation 55 ± 10, between the ages of 40 and 77) were examined using the DBT method with Siemens Mammomat Revelation (Erlangen, Germany). The patients’ left mediolateral oblique (MLO) DBT exams, which demonstrate adequate levels of pectoral muscle, were documented. In Fig. 1, the “data selection flowchart” is reviewed.

The following are the inclusion criteria:

1. Cases with imaging interpretation results categorized as BIRADS 1, 2, and 3
2. Cases categorized as BIRADS 0 but with BIRADS 1, 2, or 3 findings observed in the ultrasound examination performed during the same period as the complementary tests

The following are the exclusion criteria:

1. Images containing inadequate, blurry, or no pectoral muscle tissue
2. Imaging interpretation with suspicious masses (BIRADS 4, 5, 6)
3. Patients with a history of cancer surgery, radiation therapy, or chemotherapy
4. Patients on prescription for diabetes
5. Right MLO images: not included in the study because of the expected hypertrophy of the right pectoral muscle caused by the more frequent use of the right upper extremity in the general population compared with the left

### Study design

The left DBT images of the 40-year and older female patients included in the study who applied to Medipol Mega Hospital for comprehensive health screening were collected consecutively and anonymously in DICOM format from the radiologic information system to create a data set. The HbA1c% values obtained from the blood samples taken for diabetes screening were recorded from the electronic hospital information system. The blood samples were collected from the patients on the same day of the DBT. No additional DBT or HbA1c tests were performed for this study.

In routine practice, plasma HbA1c is the standard diagnostic test for evaluating a patient’s chronic blood sugar level. The HbA1c values of patients with normal values (≤ 5.5%) constituted the control group. Cases with HbA1c values between 5.6 and 6.4%were categorized as prediabetic and those with ≥ 6.5% as diabetic and included in the study group. Each group was divided into three subgroups according to age: 40–49 years, 50–59 years, and 60 years and above. We tried to provide the minimum number of images to maintain successful learning by the AI. Therefore, a minimum of 1000 images were aimed to be collected for each category in each age range. In total, 13,927 images were collected first. More than one image from the same patient was entered into our dataset. To achieve cross-validation, all images from a single patient were analyzed either in the training group or in the testing group. This way, we attempted to prevent overfitting.

Before feeding the AI, image selection and some narrowing of groups were performed to prevent the model’s bias and to provide equal distribution among subgroups covering 3 age ranges (40–49 years, 50–59 years, and 60 years and over) for all three categories (normal, prediabetic, and diabetic). Overall, 11,594 pectoral muscle-containing images were entered into the model. Numbers belonging to all subgroups are shown in Table 1.

**Table 1:** Modified dataset: pectoral muscle containing image numbers per subgroup

**Images per age group**	**Normal**	**Pre-diabetic**	**Diabetic**
**40–49**	*n* = 1454	*n* = 1145	*n* = 1426
**50–59**	*n* = 1454	*n* = 1454	*n* = 1025
**≥ 60**	*n* = 994	*n* = 1222	*n* = 1420
**Total**	3902	3821	3871

The pectoral muscle images were cropped to include only the pectoral muscle. The cropped images were separated into three different categories (normal, prediabetic, and diabetic) according to the patients’ HbA1c levels and saved. These three categories formed the necessary dataset to train, evaluate, and test the AI model’s ability to distinguish between the groups. Our model determines the patients’ DM status (normal, prediabetic, or diabetic) by evaluating the density of the pectoral muscle.

### Artificial intelligence model

The modified dataset was split into 85% for training and 15% for testing the model’s performance, providing a clear separation for training and evaluation. The images mentioned above were used as the initial data and increased using data augmentation techniques, as a data replication/improvement method. Data augmentation was applied using the ImageDataGenerator from Keras with horizontal and vertical flips and rotation (-10, + 10 degrees). This was specified in the datagen object: ImageDataGenerator (horizontal_flip=True, vertical_flip=True). The techniques employed increased the diversity of the dataset and impacted the model’s accuracy positively. To further detail the deep learning methods for this image classification problem, convolutional neural networks (CNN) were used. Ready-made architectures that have proven successful in this field were taken, retrained to be used in our problem, and the model weights were updated.

**Fig. 2:**
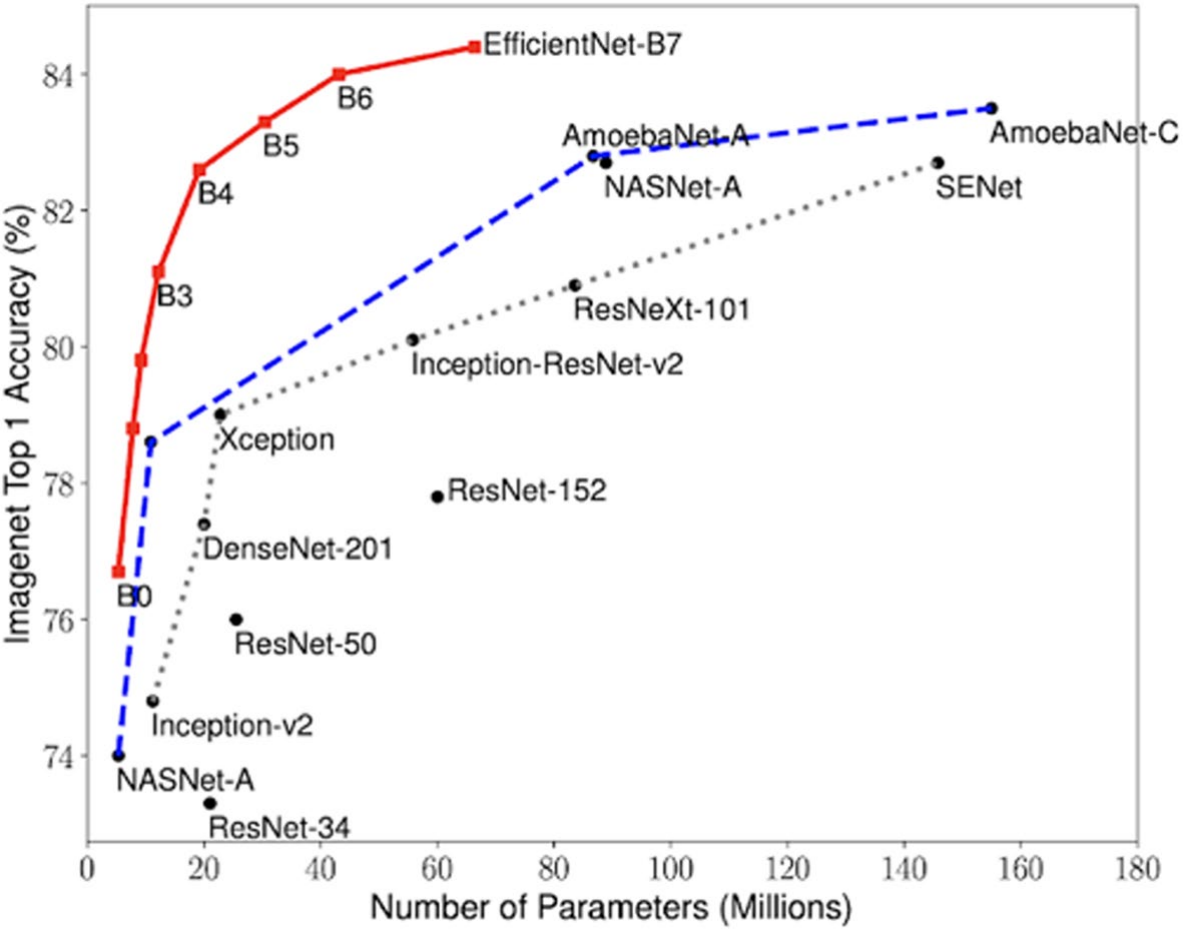
Comparison of EfficientNet and other ESA models’ success rates

The artificial neural network architecture we used was EfficientNetB5, which was adapted to our current classification problem. EfficientNet was developed by Google AI in 2019. When tested on ImageNet, a large visual database designed for visual object recognition software research, enterprise security architecture was found to produce classification models with faster and higher accuracy rates than other architectures because of its system for almost optimal scaling (Google Research, 2019). Figure 2 shows a comparison of success rates on ImageNet with other popular pre-trained architectures. As shown in this figure, EfficientNet models achieve higher accuracy and better efficiency with fewer parameters than current CNNs.

**Fig. 3:**
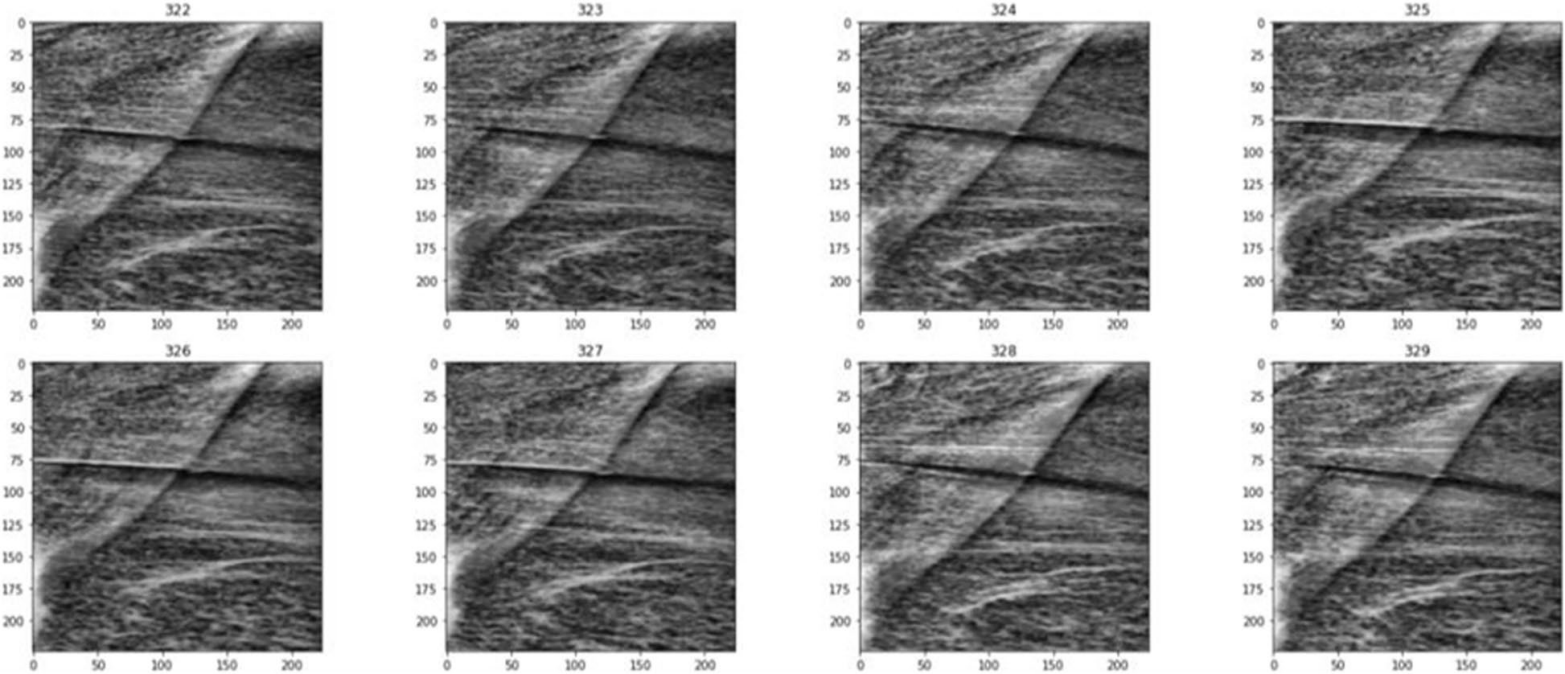
Examples of images that enter the model after cropping and resizing

The EfficientNetB5 architecture was used for transfer learning and adapted to the specific classification problem of normal, prediabetic, and diabetic classes. At the end of the architecture, the SoftMax function was chosen as the activation function for multi-class classification problems. Labeled images belonging to three classes (normal, prediabetic, diabetic) that underwent data augmentation and were resized to (224, 224) were separated into training and testing sets. Examples of images fed into the model after the cropping and resizing processes are shown in Fig. 3.

**Fig. 4:**
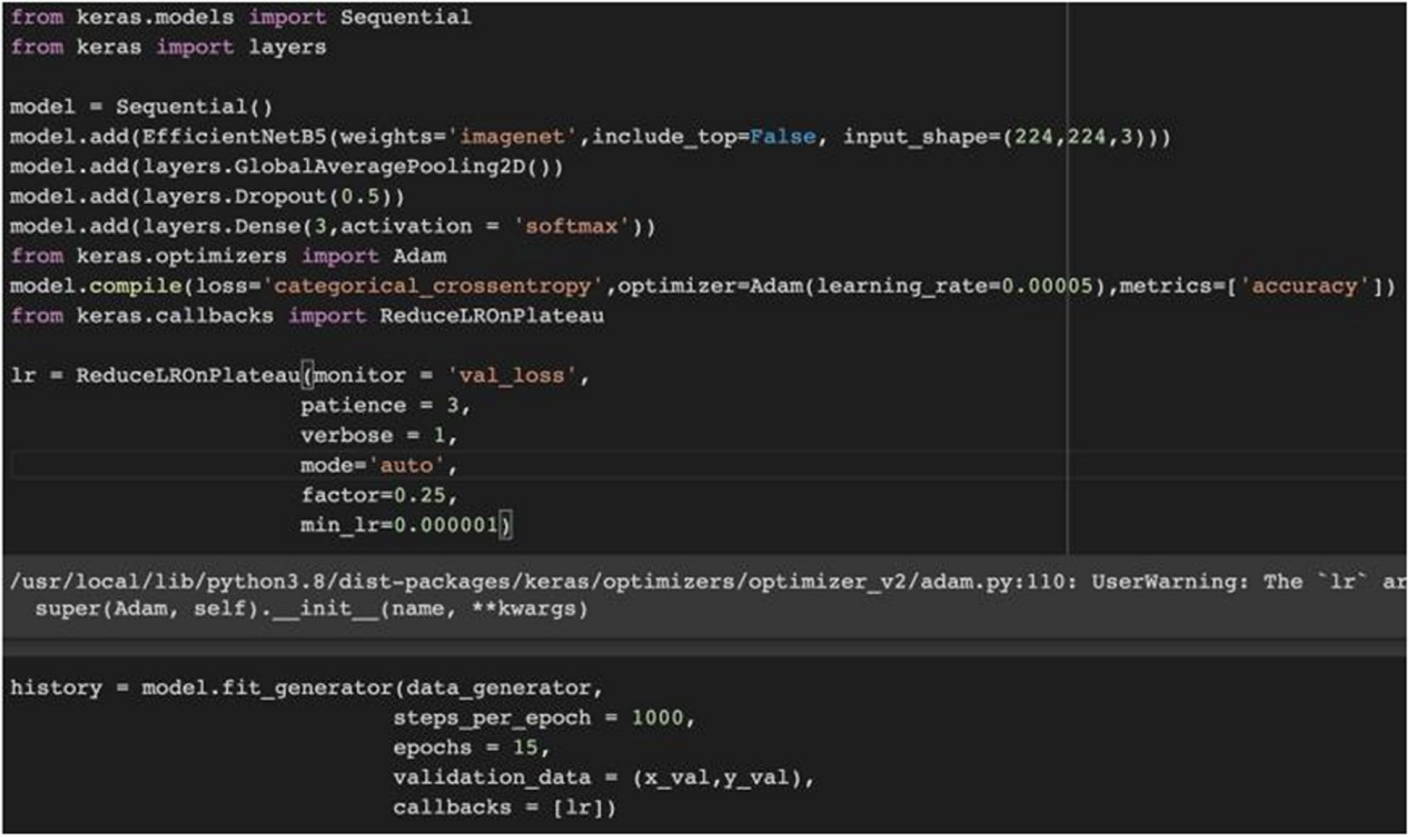
Model parameters

The model training process was initiated using the parameters shown in Fig. 4. Training parameters, including the learning rate, were selected based on the architecture’s characteristics and best practices.

**Fig. 5:**
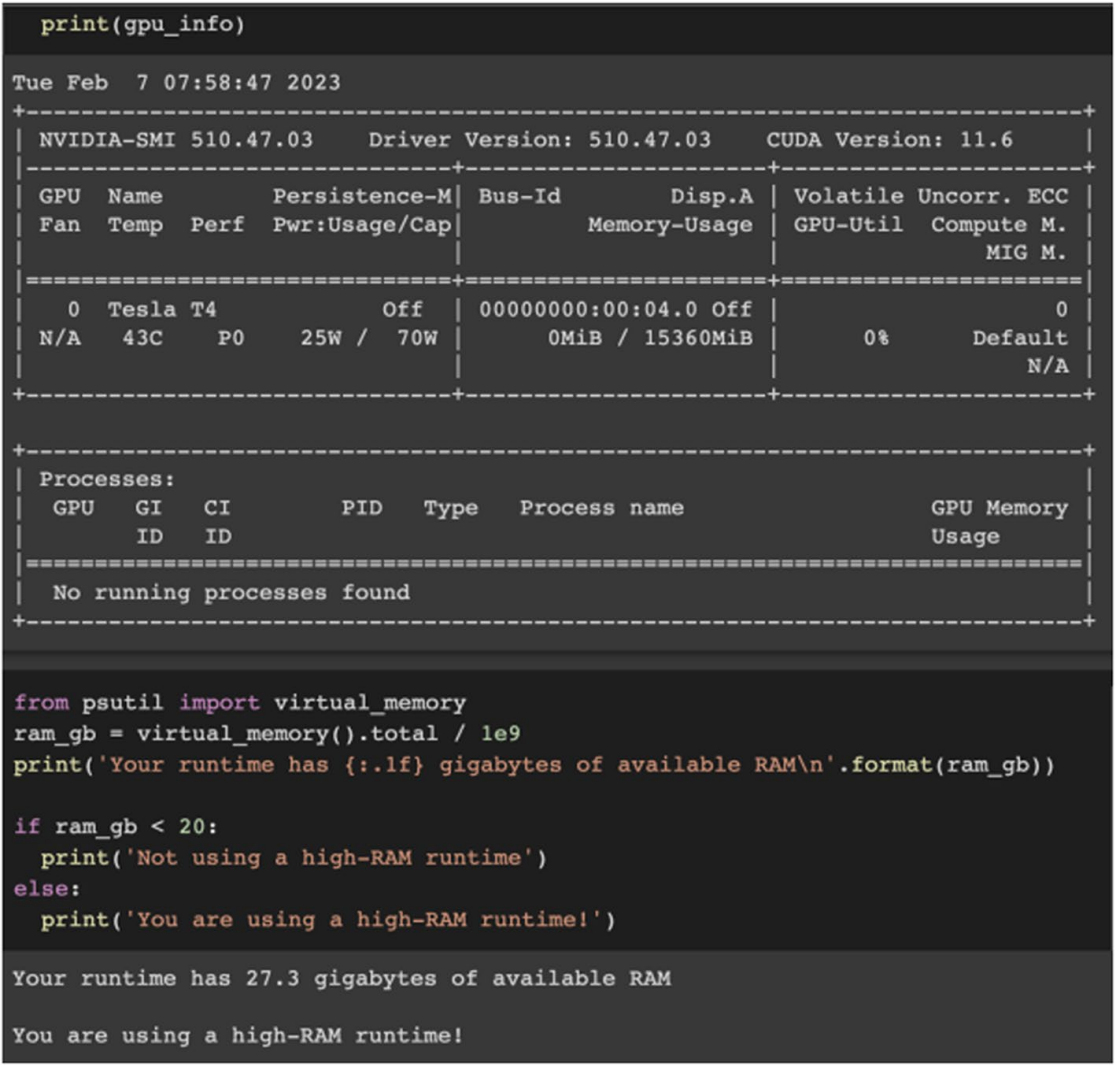
Details and information on Google Colab GPU and RAM

During the model’s training process, Google Colab was used, which is specifically designed for machine and deep learning and has professional resources to obtain fast results, including NVIDIA Tesla T4 GPU and 27.3 GB RAM (Fig. 5).

The validation process was present in the code using the validation data parameter in the model fit generator function. The validation data was provided as (x_val, y_val). However, details about external validation or a more comprehensive validation strategy were not explicitly mentioned in the provided code. The code includes the hyperparameter tuning for the learning rate (lr) using ReduceLROnPlateau. The learning rate is adjusted based on the validation loss. The code incorporates the model training, saving, and evaluation outcomes using the “Classification eport” from “scikit-learn,” providing metrics such as precision, recall, and F1-score for each class. We used these performance indicators to track the progress of our AI model. The priority was given to the accuracy rate. The accuracy rate is described as the ratio of the correctly predicted areas in the model to the total dataset. While the current code focuses on accuracy, precision, recall, and F1-score, additional metrics like area under the receiver operating characteristic curve (AUC-ROC), positive predictive value (PPV), and negative predictive value (NPV) were also calculated.

## Results

The training process of our selected model was completed in the 15th epoch, each consisting of 1000 steps, with an accuracy rate of 92% and a loss of only 0.22 (Table 2).

**Table 2:** Accuracy and loss values of the training iterations

***Epoch 15 steps per epoch = 1000*** ***Model/data fit generator*** ***iphyton input***	***ms/step***	***loss***	***Accuracy***	***Validation data loss***	***Validation accuracy***	***callbacks***
*Epoch 1/15 - 167s*	141	1.1041	0.3590	1.0632	0.4362	5.000e−05
*Epoch 2/15 - 135s*	135	1.0670	0.4340	1.0191	0.5046	5.000e−05
*Epoch 3/15 - 136s*	136	1.0043	0.5110	0.9447	0.5552	5.000e−05
*Epoch 4/15 - 136s*	136	0.9489	0.5495	0.8263	0.6282	5.000e−05
*Epoch 5/15 - 136s*	136	0.8795	0.6005	0.8034	0.6448	5.000e−05
*Epoch 6/15 - 136s*	136	0.8193	0.6320	0.6383	0.7305	5.000e−05
*Epoch 7/15 - 136s*	136	0.7507	0.6810	0.5571	0.7856	5.000e−05
*Epoch 8/15 - 136s*	136	0.6613	0.7220	0.4912	0.7897	5.000e−05
*Epoch 9/15 - 136s*	136	0.5542	0.7660	0.4433	0.8103	5.000e−05
*Epoch 10/15 - 136s*	136	0.5055	0.7975	0.3150	0.8885	5.000e−05
*Epoch 11/15 - 136s*	136	0.3943	0.8500	0.2988	0.8931	5.000e−05
*Epoch 12/15 - 136s*	136	0.3728	0.8570	0.2376	0.9224	5.000e−05
*Epoch 13/15 - 136s*	136	0.3124	0.8800	0.2040	0.9322	5.000e−05
*Epoch 14/15 - 136s*	136	0.2849	0.8925	0.1816	0.9368	5.000e−05
*Epoch 15/15 - 137s*	137	0.2202	0.9245	0.1427	0.9454	5.000e−05

**Fig. 6:**
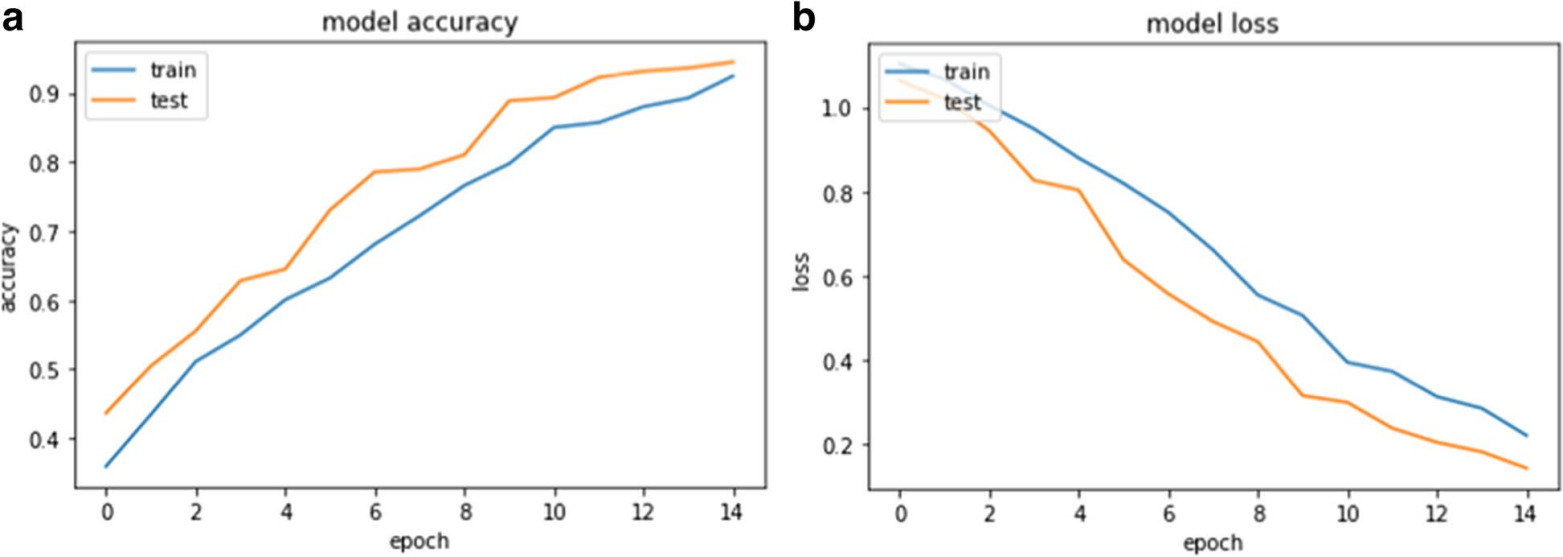
**a** Model’s accuracy. **b** Model’s loss rate after each training round

As an indicator of a healthy learning process, the parallel run of training and test sets was observed for both the models’ accuracy and loss rate*.* Figure 6a shows that the success rates for both the training and test sets increased for each training round, whereas Fig. 6b shows that the loss rates decreased at a similar and regular pace for both the training and test sets for each training round. In addition to these analyses, the F1-score, which is summarized as the harmonic mean of the precision and recall values of the models, was tracked as an auxiliary indicator since the accuracy rate alone was not considered sufficient to prove a model’s success. The AUC-ROC is 0.995. The PPV is 94%, and the NPV of our model is 98%. These metrics provide a more comprehensive evaluation of the model’s performance, indicating high discriminative ability, precision in positive predictions, and reliability in negative predictions.

The confusion matrix in Table 3 can be interpreted as follows:

**Table 3:** Confusion matrix

	**Precision (specificity)**	**Recall (sensitivity)**	**F1-score**	**Support (numbers of images)**
0 (control group)	0.98	0.90	0.94	577
1 (prediabetics)	0.93	0.95	0.94	583
2 (diabetics)	0.93	0.98	0.96	580
Accuracy			0.95	1740
Macroaverage	0.95	0.95	0.95	1740
Weighted average	0.95	0.95	0.95	1740

Precision (specificity): This indicates the percentage of images predicted by the model to belong to a certain group that were actually in that group. According to the confusion matrix, for the test set, out of 577 images in the normal group, 98% were correctly identified as normal by the model. This rate is 93% for prediabetic and diabetic patients. When the averages of the three categories are taken, the precision value of the model is 95%.

Recall (sensitivity): This indicates the percentage of images that actually belong to a certain group that were correctly predicted by the model. According to the matrix, 90% of the images belonging to the normal class were correctly identified by the model. The same rate was 95% for prediabetic cases and 98% for diabetic cases.

F1-score: The F1-score is calculated by taking the harmonic mean of the above values. For normal cases, the F1-score is 0.94, for prediabetic cases is 0.94, and for diabetic cases is 0.96. Considering that the best F-score can be at most 1, it can be concluded that the model has a high F1-score for all three categories and is valuable in predicting the images.

Support: This value indicates the number of images in each class of the test dataset.

## Discussion

Our study has shown an innovate approach using deep learning AI technology that has the potential to detect the diabetes status of women using pectoral muscle images on DBT (with an accuracy of 92%). There are several other imaging techniques for muscle tissue, including magnetic resonance imaging (MRI), computed tomography (CT), ultrasonography, bioelectrical impedance analysis, and dual-energy X-ray absorptiometry [13, 14]. The use of cross-sectional imaging, such as CT and MRI, in modern medicine is widespread and sometimes regarded as the gold standard for assessing the body’s proportions of muscle and fat [15]. Moreover, CT has aimed to be used as an opportunistic screening modality for various conditions beyond the desired clinical indication and is a potentially useful modality for diagnosing type 2 diabetes mellitus, but has not yet been used as such [16]. Yet their high cost, limited accessibility, high-dose radiation exposure (in CT), and labor-intensive segmentation procedure limit their usage in clinical research [17]. To overcome these shortcomings, we took advantage of muscle images obtained from DBT to creatively enhance its use beyond breast tissue evaluation as an opportunistic screening tool for T2DM. Further studies can be conducted on comparing the performance of DBT with the other modalities for their ability to assess muscle tissues.

The opportunistic nature of our model holds immense potential in targeting a susceptible group, as screening of women older than 40 years has already been recommended by the World Health Organization every 2 years. The development of our AI model offers a non-invasive alternative method to blood biochemistry, with several notable advantages (Table 4). Our model demonstrates the same accuracy as the standard diagnostic method, highlighting its reliability and potential for clinical implementation. Moreover, it enables a more rapid diagnosis, thereby reducing the time required for healthcare professionals to assess patients. By employing an AI-based approach, our method can minimize the need for extensive healthcare professional involvement and may be profitable and practical for widespread adoption.

**Table 4:** DM diagnosis via routine blood chemistry versus digital breast tomosynthesis (DBT)-based artificial intelligence in women

	**Blood chemistry**	**DBT-AI**
**Method**	Invasive	Non-invasive
**Time**	8–24 h	10–15 min
**Accuracy**	92.5 ± 6%^8^	92%
**Workload**	Doctor, nurse, lab technician	Radiology technician, AI model
**Indication**	Symptomatic patients	Opportunistic diagnostic tool for comprehensive health screening of postmenopausal patients

One significant implication of our study is the potential expansion of our algorithm for diagnosing muscle tissue-related conditions beyond diabetes mellitus. The model can be further developed to derive additional diagnostic information from images containing muscle tissue. By leveraging the algorithm’s capability to analyze various image textures, it may be applied as a diagnostic tool for disorders such as Duchenne/Becker dystrophy, rhabdomyosarcoma, dermatomyositis/polymyositis, and congenital metabolic storage diseases. This expansion could benefit patients and healthcare professionals alike, offering a non-invasive and efficient approach to diagnosing a range of conditions. To comprehensively diagnose diseases and manage treatment options, supplementary studies are recommended for developing a versatile deep learning system that combines various modalities of information, such as patient symptoms, imaging from different modalities, blood test data, and clinical data [18].

Additionally, the relationship between insulin and lower breast density in premenopausal women is known. According to a study, diabetes mellitus could potentially affect breast density in DBT through the insulin signaling pathway [19]. In light of this information, in a different study design, our dataset can be used for screening women with confirmed diabetes mellitus in terms of breast cancer risk. On a technical level, another study strength is the use of convolutional neural networks (CNN) to train our AI model. CNN, which originated from the deep learning community, have been adopted by the radiological community because of their ability to learn spatial features from medical images [20].

Despite these strengths, our study has certain limitations that should be acknowledged. First, there may be potential population bias because the study’s patient selection might not fully represent the diverse demographics of the general population. Second, the retrospective design of our study may introduce inherent limitations and biases in data collection and analysis. Third, the limited number of patients included in our study might restrict the generalizability of our findings. It is important to conduct further research with larger and more diverse patient groups to validate and refine our AI model. Moreover, our study acknowledges the limitation of multiple images from a single patient, which could introduce correlations or dependencies within the dataset. This issue should be carefully considered when interpreting the results and further developing the algorithm. Lastly, our study focuses primarily on women, which limits the generalizability of our findings to the broader population. Future studies should include a more balanced representation of both genders to ensure the applicability of our AI model across diverse populations.

In conclusion, our study showed that AI can detect diabetes mellitus status from the pectoral muscle on DBT images, which could be an inspiration for other studies focusing on AI development.

## Data Availability

The imaging data used and/or analyzed in this study are available from the corresponding author upon reasonable request.

## Abbreviations

AI: Artificial intelligence
CNN: Convolutional neural networks
LMO: Left mediolateral oblique
T2DM: Type 2 diabetes mellitus
